# Functional Characterization of Tomato *Phytochrome A* and *B1B2* Mutants in Response to Heat Stress

**DOI:** 10.3390/ijms23031681

**Published:** 2022-01-31

**Authors:** Islam M. Y. Abdellatif, Shaoze Yuan, Renhu Na, Shizue Yoshihara, Haruyasu Hamada, Takuya Suzaki, Hiroshi Ezura, Kenji Miura

**Affiliations:** 1 Graduate School of Life and Environmental Sciences, University of Tsukuba, Tsukuba 305-8572, Japan; islam_abdellatif@mu.edu.eg (I.M.Y.A.); yuanshaoze1994@yahoo.co.jp (S.Y.); na.renhu.gb@un.tsukuba.ac.jp (R.N.); suzaki.takuya.fn@u.tsukuba.ac.jp (T.S.); ezura.hiroshi.fa@u.tsukuba.ac.jp (H.E.); 2 Department of Horticulture, Faculty of Agriculture, Minia University, El-Minia 61517, Egypt; 3 Department of Biological Science, Osaka Prefecture University, Sakai 599-8531, Japan; yoshihara@b.s.osakafu-u.ac.jp; 4 Pharma and Supplemental Nutrition Solutions Vehicle, Kaneka Corporation, Iwata 438-0802, Japan; haruyasu.hamada@kaneka.co.jp; 5 Tsukuba-Plant Innovation Research Center, University of Tsukuba, Tsukuba 305-8572, Japan

**Keywords:** tomato, PHYTOCHROME A, PHYTOCHROME B1B2, *phyA*, *phyB1B2*, heat tolerance, HS

## Abstract

Heat stress (HS) is a prevalent negative factor affecting plant growth and development, as it is predominant worldwide and threatens agriculture on a large scale. PHYTOCHROMES (PHYs) are photoreceptors that control plant growth and development, and the stress signaling response partially interferes with their activity. PHYA, B1, and B2 are the most well-known PHY types in tomatoes. Our study aimed to identify the role of tomato ‘Money Maker’ *phyA* and *phyB1B2* mutants in stable and fluctuating high temperatures at different growth stages. In the seed germination and vegetative growth stages, the *phy* mutants were HS tolerant, while during the flowering stage the *phy* mutants revealed two opposing roles depending on the HS exposure period. The response of the *phy* mutants to HS during the fruiting stage showed similarity to WT. The most obvious stage that demonstrated *phy* mutants’ tolerance was the vegetative growth stage, in which a high degree of membrane stability and enhanced water preservation were achieved by the regulation of stomatal closure. In addition, both mutants upregulated the expression of heat-responsive genes related to heat tolerance. In addition to lower malondialdehyde accumulation, the *phyA* mutant enhanced proline levels. These results clarified the response of tomato *phyA* and *phyB1B2* mutants to HS.

## 1. Introduction

Abiotic stress caused by environmental changes negatively affects plant growth and development [1]. Global warming has greatly impacted agriculture [2], and in many areas of the world, heat stress (HS) is one of the most crucial threats to plant growth and development, as it leads to a severe reduction in economic yield by causing morpho-anatomical, physiological, and biochemical changes in plants [3]. From 2050 to 2100, HS will likely negatively affect tomato growth and productivity in the open field and decrease the optimal areas for cultivation [4]. An approach to mitigate the adverse effects of HS is to develop crop plants with improved plant stress tolerance using various genetic methods [3].

Tomato (*Solanum lycopersicum* L.) is an economically important crop worldwide that is sensitive to a series of abiotic stresses, particularly extreme temperatures [5]. The optimum temperature for tomato growth, fruit set, and yield ranges between 21 and 29.5 °C during the day and between 18.5 and 21 °C during the night [6].

PHYTOCHROMES (PHYs), which absorb red and far-red light, are the most characterized photoreceptors in plants [7]. Plant growth and development (from seed germination to flowering) can be controlled by PHYs. PHYs regulate both biotic stress and stress induced by abiotic factors, such as high and low temperatures, salinity, drought, toxic metals, ultraviolet B radiation, and herbivory [8], by changing a wide range of biochemical and molecular responses [7]. The number and types of PHYs vary among plant species. Tomato and *Arabidopsis* contain five *PHYs* in their genomes. Tomato plants have *PHYA*, *PHYB1*, *PHYB2*, *PHYE*, and *PHYF* [9], whereas *Arabidopsis* contains *PHYA*–*PHYE* [10]. In rice, plants have only three *PHYs*, *PHYA*–*PHYC* [11]. Bioinformatics analysis of microarrays has clarified the performance of *Arabidopsis* PHYA and PHYB photoreceptors in plant HS responses [12] and revealed that PHYB also functions as a photoreceptor and temperature sensor in *Arabidopsis* plants [13].

Previous studies have shown that *phy* mutants are tolerant to several abiotic stresses. *Arabidopsis phyB* mutants exhibit tolerance to HS by lowering the percentage of electrolyte leakage (EL) [14]. High evapotranspiration levels caused rapid wilting in tomato *phyA* mutants compared to wild-type (WT) plants [15,16], which was also observed after exposure to sunny summer days [17]. In addition, rice *phyB* mutants exhibit improved cold tolerance compared to WT plants because of their lower EL index and malondialdehyde (MDA) concentration [18]. Furthermore, under salinity stress, tobacco *phyA*, *phyB,* and *phyAB* mutants exhibited greater tolerance than WT plants by lowering their EL and MDA accumulations as well as enhancing the transcript levels of their defense-associated genes and the activities of some antioxidant enzymes [19].

Generally, plants can adapt to environmental conditions by developing a dynamic response at the morphological, physiological, and biochemical levels [20], in addition to enhancing specific changes in their gene expression levels and metabolism [21]. Under HS conditions, plants modulate their physiological function to respond or adapt to temperature changes [22]. Cell membrane injury is one of the first effects of plant stress. Maintaining electrolytes and stability of the cell membrane are important components for plant tolerance to stress conditions, such as high-temperature stress [23]. Proline is an essential organic osmolyte [24]. Its accumulation is a common physiological response to a wide range of biotic and abiotic stresses [25], such as extreme temperatures [24], and it is associated with stress tolerance [26]. Membrane lipid peroxidation is a severe damaging effect of reactive oxygen species (ROS), and measuring MDA can detect this condition, which is an important indicator of oxidative lipid injury caused by environmental stress. Several studies have investigated the MDA levels in plants under different stress conditions [27]. Stomata are microscopic pores in the leaf epidermis that regulate transpiration and carbon dioxide uptake by plant leaves. The guard cells of stomata can sense various stress triggers and respond quickly to initiate closure under unfavorable conditions [28].

For plant molecular mechanisms, HEAT SHOCK TRANSCRIPTION FACTORS (HSFs) and HEAT SHOCK PROTEINS (HSPs) are important factors in HS response and the acquisition of heat tolerance in plants [29,30]. The HSFs involved in heat sensing and signaling conserve their structure and functionality throughout the eukaryotic kingdom and are main triggers for the expression of *HSP* genes [29]. Studies have identified and characterized the *HSF* gene family in many crop species, including tomatoes [31]. Strong induction occurs in response to HS, with stable expression levels under nonstressed conditions [29]. The production and activation of HSFs and HSPs play a key role in plant response and acclimation to HS [32,33,34].

High temperature directly affects tomato fruit setting [35] because of its negative influence on pollen release and viability, which are major factors limiting fruit set [36]. In addition, high temperatures increase the production of aborted flowers, poor flower fertilization, and parthenocarpic fruit [37].

Many studies have illustrated the role of *PHYB* in abiotic stresses such as those relating to light, heat, cold, drought, and salinity, while only a few have described the role of *PHYA* under these conditions. An ideal tool for studying the participation of *PHYs* in biotic and abiotic stress responses is to increase the availability of *phy* mutants in different plant species [38]. Therefore, this study aimed to identify the response of tomato *PHYs* A and B to HS during different growth stages using the *phyA* mutant and the *phyB1B2* double mutant.

## 2. Results

### 2.1. The Phenotype of phy Mutants in Response to HS

#### 2.1.1. Conservation of the Seed Germination Rate in *phyA* and *phyB1B2* under HS

**Figure 1:**
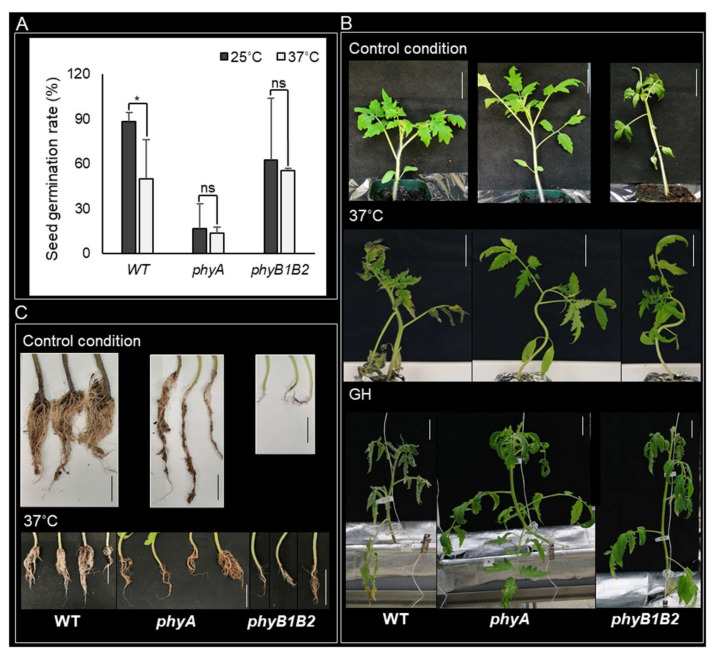
Plant features during seed germination and vegetative growth stages under heat stress (HS). (**A**) The seed germination rate of WT, *phyA*, and *phyB1B2* after 7 days at 25 or 37 °C. (**B**) The phenotype of 4-week-old plants of tomato WT and *phyA* and *phyB1B2* mutants under control conditions at 25 °C and after exposure to HS at 37 °C or fluctuating high temperature in greenhouse (GH) conditions for 2 weeks. (**C**) Root phenotype of 4-week-old plants of WT and *phyA* and *phyB1B2* mutants after 2 weeks under control conditions at 25 °C or HS at 37 °C. The scale bars represent 5 cm. Values represent the means ± SD (n ≥ 10) from a representative of three biological replicates. The asterisk symbol (*) represents statistically significant differences (*p* < 0.05), while ns represents statistically nonsignificant differences, between each genotype individually under 25 and 37 °C according to one-way ANOVA with post hoc Tukey HSD test.

HS can reduce the seed germination potential, resulting in poor germination and stand establishment [39]. We studied the response of the *phy* mutants to HS at the seed germination stage by measuring the seed germination rate of the WT and the mutants at 25 and 37 °C after incubation for one week. At 25 °C, *phyA* showed a lower germination rate than WT and *phyB1B2*, with similar results at 37 °C. However, *phyA* did not show any marked difference at 37 °C compared to the results at 25 °C. In addition, HS did not affect the germination rate of *phyB1B2* compared to that at 25 °C, showing nonsignificant variance between both conditions. In contrast, the WT revealed a significant reduction in seed germination rate under HS conditions compared to that at 25 °C (Figure 1A). These results indicate that HS did not affect seed germination of *phyA* and *phyB1B2* at 37 °C for 7 days.

#### 2.1.2. Enhancement of Tomato Tolerance toward HS by *phyA* and *phyB1B2* Mutation

HS enhanced several morphological symptoms including scorching and sunburns of leaves and branches, as well as leaf senescence and abscission [40]. To investigate the response of tomato *phy* mutants to HS, we grew WT, *phyA,* and *phyB1B2* under control (25 °C) and HS conditions (37 °C and greenhouse (GH)). The GH minimum recorded temperature was approximately 20 ± 3 °C while the maximum temperature was approximately 57 ± 3 °C (Figure S1). The tomato cultivar ‘Money Maker’ was used as the WT. One-month-old plants exhibited healthy growth under control conditions. After exposing all genotypes to 37 °C, the WT leaves were severely damaged compared to those of *phyA* and *phyB1B2*. Furthermore, under GH conditions, *phyA* plants were tolerant to HS, whereas WT plants exhibited weak phenotypic growth with leaf senescence, and the *phyB1B2* plants exhibited improved growth compared to the WT plants (Figure 1B). These results indicate that *phy* mutants promoted plant phenotypic tolerance to high-Tm stress.

In addition, we observed the root phenotype of WT and *phy* mutants grown at 25 and 37 °C. The WT root phenotype was more prolific than the *phy* mutant roots under both conditions (Figure 1C). This suggests that PHYA and B genes may be involved in root growth, since Arabidopsis *phyAB* mutant seedlings showed a reduction in root elongation compared to WT [41].

#### 2.1.3. The *phyA* and *phyB1B2* Flower and Fruit Phenotypic Response under HS

**Figure 2:**
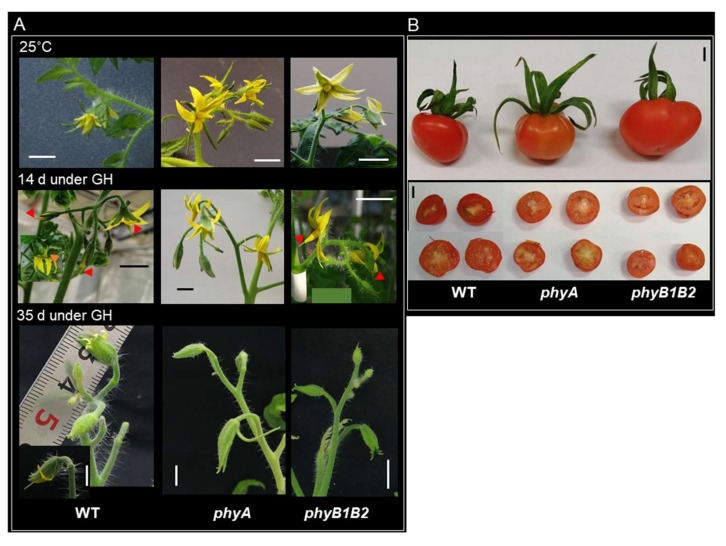
Flower and fruit phenotypic response under heat stress (HS). (**A**) The flower phenotype of WT, *phyA*, and *phyB1B2* under 25 °C and high-temperature stress under greenhouse (GH) conditions for 14 or 35 days. (**B**) Fruit phenotype and parthenocarpic phenomena of WT, *phyA*, and *phyB1B2* under GH conditions. The scale bar represents 1 cm.

Tomato flowers are highly susceptible to HS. High Tm can cause some morphological changes in the flower structure [42]. The flower phenotype was observed in flowered plants exposed to fluctuating high temperature in the GH for 14 and 35 days in comparison with flowers formed under 25 °C. The flowers of all genotypes did not show any marked difference under 25 °C. HS application for 14 days changed the flower phenotype of either WT or *phyB1B2* compared to control conditions. At the same time, there was no difference in *phyA* flowers under either control or HS conditions. On the other hand, all genotypes showed different flower phenotype when exposed to HS for more than one month compared to plants under HS for two weeks. In *phyA,* the sepaloid was the dominant part of the flowers showing long sepal. In addition, bloomed flowers did not appear under a long HS period (Figure 2A). These results show that HS affects flowers of WT and *phy* mutants after a long exposure time in a similar way.

High Tm enhanced the parthenocarpic phenomena in tomato fruits [37]. Tm at 26 °C promoted small parthenocarpic tomato fruits, leading to low fruit yield [43]. The fruit phenotype and seed formation in the WT and *phy* mutants were investigated under GH conditions. The fruit phenotype of all genotypes did not show any difference. Furthermore, WT, *phyA*, and *phyB1B2* produced parthenocarpic fruits under HS (Figure 2B). These results indicate that HS negatively affected WT, *phyA*, and *phyB1B2* during the fruiting stage.

#### 2.1.4. Changes in Plant Vegetative Characteristics of *phy* Mutants in Response to HS 

High Tm adversely influences plant growth and development [44]. We measured the root surface area, length, stem height, stem thickness, plant fresh weight (FW), leaf number/plant, and average FW of leaves of one-month-old plants of WT and *phy* mutants after 2 weeks at 25 or 37 °C. The root surface area was highly significant in the WT compared to the *phy* mutants under both conditions. In addition, the *phyB1B2* root surface area was significantly lower than that of *phyA* under normal conditions, but not under HS conditions (Figure S2A).

**Figure 3:**
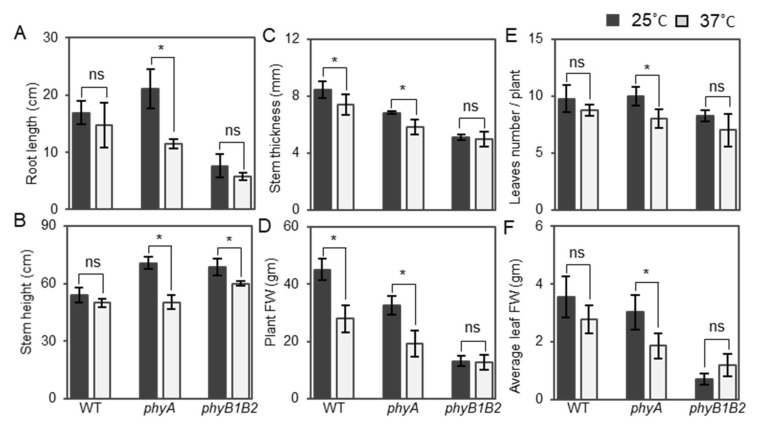
Root length and plant vegetative characteristics under heat stress (HS). Root length (**A**), plant stem height (**B**), stem thickness (**C**), plant FW (**D**), number of leaves/plant (**E**), and average leaf FW (**F**) of WT and *phyA* and *phyB1B2* mutants after exposing 4-week-old plants to HS conditions at 37 °C for 2 weeks in comparison with control conditions at 25 °C. Values represent the means ± SD (n = 4) from a representative of three biologically independent experiments. The asterisk symbol (*) represents statistically significant differences, while ns represents statistically nonsignificant differences, between each genotype individually under control and HS conditions according to Duncan’s test (*p* < 0.05).

The *phyA* mutant exhibited a long root length, which was not significantly different from that of WT, but differed significantly from *phyB1B2* under control conditions (Figure S2B); however, the length significantly decreased after HS application compared to that at 25 °C. In contrast, there was no observable significant difference in the root length of WT or *phyB1B2* at 25 and 37 °C (Figure 3A).

WT plants did not exhibit any marked differences in stem height, leaf number/plant, and average FW of leaves under both conditions, whereas there was a significant reduction in stem thickness and plant FW under HS conditions compared to the control. *PhyA* plants exhibited a significant reduction in all plant vegetative characteristics, such as stem height, stem thickness, plant FW, leaf number/plant, and average leaf FW at 37 °C compared to 25 °C. In contrast, no difference was observed among all vegetative parameters, except for plant stem height in *phyB1B2* (Figure 3B–F). Based on these results, the *phyA* mutant regulated the growth and enhanced the reduction of growth parameters at 37 °C while the *phyB1B2* mutant recorded lower values in most of the vegetative growth parameters.

#### 2.1.5. Tomato *phyA* Mutant Inhibited Undesirable Flower Traits under HS

High Tm causes undesirable defects in tomato flowers. There are indicated parameters related to floral characteristics that are used to evaluate tomato tolerance to HS in the flowering stage, such as stigma exertion, antheridia cone splitting, and flower number [45]. HS induced stigma exertion and antheridia cone splitting [46,47].

**Figure 4:**
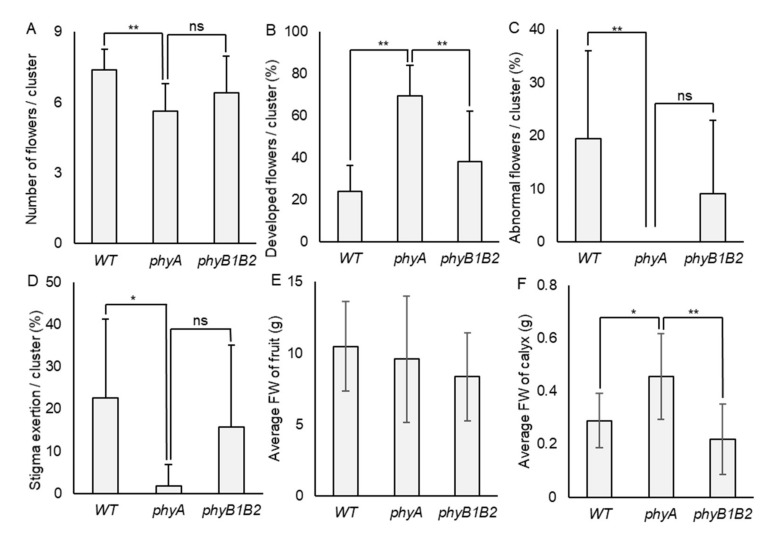
Flower and fruit characteristics under heat stress (HS). (**A**) The number of flowers/cluster, (**B**) the percentage of developed flowers/cluster, (**C**) the percentage of abnormal flowers/cluster (that showed antheridia cone splitting), and (**D**) the percentage of stigma exertion/cluster were detected in WT, *phyA*, and *phyB1B2* plants exposed to fluctuating high temperature for 14 days during flowering stage. (**E**) The average FW of fruit and (**F**) the average FW of calyx of WT, *phyA*, and *phyB1B2* were recorded under greenhouse (GH) conditions. The average temperature is recorded in Figure S1. The minimum Tm was 17.0 °C while the maximum was 50.5 °C. Data represent the means ± SD (n ≥ 8). The asterisk symbol (*) represents statistically significant differences (*p* < 0.05), double asterisk symbol (**) expresses statistically highly significant differences (*p* < 0.01), and ns abbreviation represents statistically nonsignificant differences between all genotypes under HS conditions according to one-way ANOVA with post hoc Tukey HSD test.

To determine the tolerance of *phy* mutants toward HS at the flowering stage, all genotypes endured fluctuating high temperatures in the GH for 14 days, where the minimum temperature reached 17.0 °C and the maximum reached 50.5 °C during flower formation (Figure S1). We recorded the total number of flowers/cluster, number of developed flowers/cluster, percentage of abnormal flowers/cluster, and percentage of stigma exertion/cluster. Under HS conditions, the WT achieved a higher number of flowers/cluster compared to *phyA*, which was highly significant. At the same time, there was no marked variance between WT and *phyB1B2*. The number of developed flowers/cluster showed a significant increase in *phyA* compared to WT and *phyB1B2*. The difference in the percentage of abnormal flowers/cluster between WT and *phyA* was also highly significant (*p* < 0.01). *PhyA* showed a significantly lower percentage of abnormal flowers compared to WT, while they were not significantly different when compared to *phyB1B2*. *PhyA* revealed a significantly lower percentage of stigma-exerted flowers compared to WT plants, with no significant difference compared to *phyB1B2*. These results indicate that *phyA* is tolerant to high-temperature stress during the flowering phase for a few days of HS treatment, as opposed to WT and *phyB1B2*. Although *phyB1B2* did not show a marked difference from *phyA*, it was still not significantly different from the WT plants (Figure 4A–D).

#### 2.1.6. Negative Effects of HS on Fruit Characteristics

High Tm adversely affects fruit characteristics. Growth Tm at 29 °C significantly decreased tomato fruit weight and seed number/fruit compared to 25 °C conditions [48]. We investigated the fruit characteristics of the WT and *phy* mutants under HS conditions, including the average FW of fruit and calyx. The average FW of fruit was affected by HS in all genotypes, with no significant difference among them (Figure 4E). The calyx FW in *phyA* was significantly increased (*p* < 0.05) compared to WT and was significantly (*p* < 0.01) higher than that of *phyB1B2* (Figure 4F). These results indicate that HS negatively affected WT, *phyA*, and *phyB1B2* during the fruiting stage.

### 2.2. Tomato phyA and phyB1B2 Physiological Response under HS Conditions

#### 2.2.1. Inhibiting EL and MDA Accumulation and Enhancing Proline Accumulation under HS by Tomato *phyA* mutation

**Figure 5:**
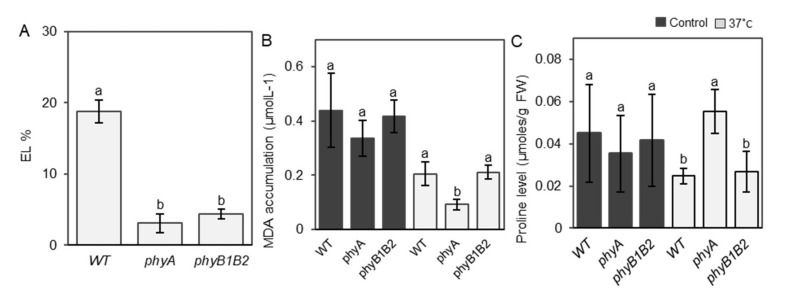
Electrolyte leakage (EL), MDA accumulation, and proline level under heat stress (HS). (**A**) The leaf EL of WT, *phyA*, and *phyB1B2* after 2 weeks of HS at 37 °C. Values represent the means ± SD (n = 6) from a representative of three biologically independent experiments. MDA accumulation (**B**) and proline level (**C**) of WT and *phyA* and *phyB1B2* mutants under 25 and 37 °C. Data represent the means ± SD (n ≥ 4) from a representative of three biologically independent experiments. The letters written on the top of the error bars show the statistically significant differences between WT and *phy* mutants under HS conditions for EL parameter and under control and HS conditions individually for MDA and proline parameters, according to Duncan’s test (*p* < 0.05). The same letter indicates no significant difference.

Membrane thermostability is a reliable parameter for screening plant tolerance to HS [49], and we determined the membrane stability of the *phy* mutants by measuring the EL of the tomato leaves. The EL of *phyA* grown at 37 °C was the lowest among all genotypes, and the value for *phyB1B2* was also significantly lower than that of WT (Figure 5A). HS reduced the EL of *phyA* and *phyB1B2* by approximately 76% and 52%, respectively, compared to that of the WT. These results suggest that *phyA* and *phyB1B2* enhance membrane thermostability.

MDA is an end product produced as a result of polyunsaturated fatty acid peroxidation in cells and is a marker of oxidative stress [50]. MDA accumulation was not significantly different between WT and the *phy* mutants under control conditions, whereas *phyA* exhibited a significant reduction in MDA levels under HS compared to WT and *phyB1B2* (Figure 5B).

Proline is an indicator of abiotic stress imposed on plants [51] and contributes to the stabilization of the subcellular structure and scavenging of free radicals [52]. At 25 °C, proline levels of all genotypes were not significantly different, whereas at 37 °C, *phyA* achieved the highest proline value, which increased by approximately 42% and 52% in comparison with WT and *phyB1B2*, respectively (Figure 5C). These results suggest that the inhibition of membrane lipid peroxidation and accumulation of proline caused the heat tolerance phenotype of the *phyA* mutant.

#### 2.2.2. Tomato *phyA* and *phyB1B2* Can Change Stomata Features under HS Conditions

**Figure 6:**
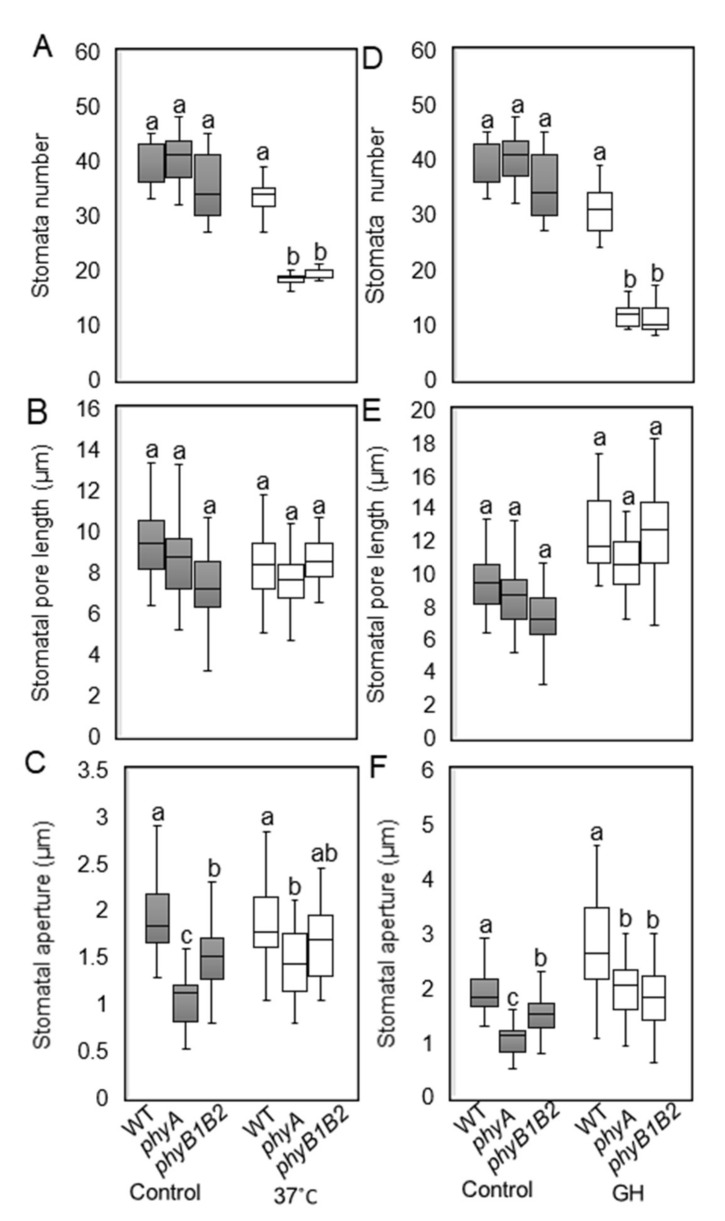
Microscopic analysis of stomata features under heat stress (HS). Stomata number/92.7mm^2^ (**A**,**D**), stomatal pore length (μm) (**B**,**E**), and stomatal aperture (μm) (**C**,**F**) after 2 weeks under HS at 37 °C and greenhouse (GH) conditions were investigated in comparison with control conditions at 25 °C. The boxplot values represent the recorded data (n ≥ 10) from a representative of three biologically independent experiments. The letters written on the top of the boxplots show the statistically significant differences between WT and *phy* mutants under control and HS conditions individually according to Duncan’s test (*p* < 0.05). The same letter indicates no significant difference.

Stomata are small pores in the above-ground organs of plants that facilitate the exchange of gases and water between plants and the surrounding environment. Their development is highly sensitive to environmental fluctuations, such as temperature stress [53]. We measured stomata number, stomatal pore length, and stomatal aperture of all genotypes grown at 25 °C, 37 °C, and GH conditions. The stomata numbers of WT and the *phy* mutants did not show significant variance under control conditions, while at 37 °C and GH conditions, WT plants contained a significantly higher number of stomata compared to the *phy* mutants (Figure 6A,D). There was no significant difference in stomatal pore length of WT and *phy* mutants under control and HS conditions (Figure 6B,E). The stomatal aperture was significantly smaller in the *phy* mutants than in the WT under control conditions. The *phyA* mutant exhibited a significant reduction in stomatal aperture compared to WT plants grown at 37 °C with no significant difference from *phyB1B2* (Figure 6C). Under GH conditions, the stomatal apertures of the *phy* mutants were significantly smaller than those of the WT plants (Figure 6F). These results suggest that the *phy* mutants may decrease water loss when under HS by reducing the number of stomata and stomatal apertures.

#### 2.2.3. Enhancing Pollen Viability under HS in Tomato *phyA* and *phyB1B2*

**Figure 7:**
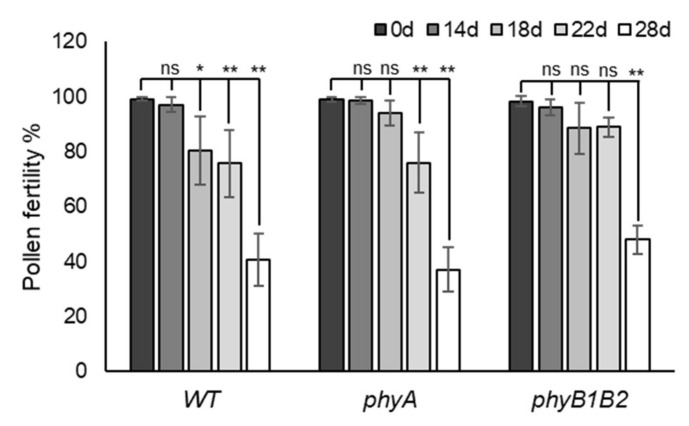
Pollen fertility under heat stress (HS). The pollen fertility percentage of WT, *phyA*, and *phyB1B2* after 0, 14, 18, 22, and 28 days of HS under greenhouse (GH) conditions. Data represent the means ± SD (n = 4). The asterisk symbol (*) represents statistically significant differences (*p* < 0.05), double asterisk symbol (**) expresses statistically highly significant differences (*p* < 0.01), and ns abbreviation represents statistically nonsignificant differences between each genotype individually after 0, 14, 18, 22, and 28 days of HS according to one-way ANOVA with post hoc Tukey HSD test.

Pollen viability is a direct test for analyzing thermotolerance in plants during the flowering stage [35]. The pollen fertility test indicates the presence of viable or fertile pollen grains. The results of pollen fertility clarified that WT, *phyA*, and *phyB1B2* differed in pollen tolerance to HS by showing different fertility percentages according to the period of HS application (Figure S3). In WT plants, the pollen fertility percentage significantly (*p* < 0.05) decreased after 18 days of exposure to fluctuating high temperatures under GH conditions. Similarly, the fertility of WT pollen after 22 and 28 days of HS had highly significant (*p* < 0.01) reductions compared to control conditions without any marked difference between pollen fertility of WT after 0 and 14 days of HS exposure. The pollen of *phyA* did not show any marked difference in fertility at 0, 14, or 18 days of HS, while it revealed a highly significant decrease after 22 and 28 days of HS. The *phyB1B2* mutant showed a nonsignificant variance in pollen fertility after application of HS for 14, 18, and 22 days compared to that at 0 days. However, there was a highly significant reduction in pollen fertility after 28 days of HS compared to that at 0 days (Figure 7). These results indicate that *phyB1B2* pollen can survive under HS for a longer time, followed by *phyA* pollen when compared to WT under HS.

#### 2.2.4. Inhibiting of Pollen Tube Growth by HS

High Tm exceeding 30 °C damages tomato pollen germination and pollen tube growth under in vitro and in vivo conditions [54]. We observed the pollen tube germination 24 h after in vivo hand pollination of the stigma at 25 °C. The results of in vivo pollen tube growth showed that WT and *phy* mutants germinated pollen after HS treatment of up to 28 days. However, few pollen tubes germinated under HS compared to those at 0 days (Figure S4). Based on these results, *phy* mutants showed the same response to high Tm stress on pollen tube growth as the WT.

### 2.3. Tomato phyA and phyB1B2 Molecular Response under HS Conditions

#### 2.3.1. Tomato *phyA* and *phyB1B2* Enhanced the Expression of Some Heat- and Stress-Responsive Genes under HS during the Vegetative Growth Stage

**Figure 8:**
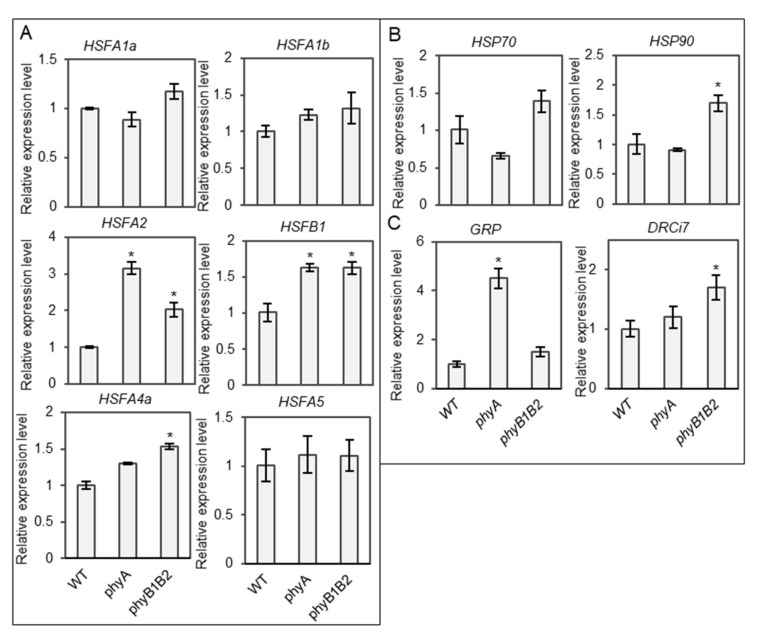
Relative gene expression levels under heat stress (HS) during vegetative growth stage. The expression levels of *HSFs* (*HSFA1a*, *HSFA1b*, *HSFA2*, *HSFB1, HSFA4a*, and *HSFA5*) (**A**), *HSPs* (*HSP70* and *HSP90*) (**B**), and stress-responsive genes (*GRP* and *DRCi7*) **(C)** of one-month-old plants of WT and *phyA* and *phyB1B2* mutants after 2 weeks under HS at 37 °C. The relative expression level of the WT was normalized to 1. Error bars represent standard deviation (*n* = 3). The asterisk symbol (*) represents statistically significant differences between WT and *phy* mutants using Duncan’s test (*p* < 0.05).

HS reduces the efficiency of plant physiological and biochemical functions by modulating molecular mechanisms [55,56,57]. HSFs and HSPs play an important role in the capability to withstand several abiotic stresses such as HS [58,59]. We examined the expression levels of heat-responsive genes, including *HSFs* and *HSPs* in WT, *phyA*, and *phyB1B2* at 37 °C. The expression levels of several *HSF* genes, such as *HSFA2* and *HSFB1*, were upregulated in both the *phyA* and *phyB1B2* mutants, and *HSFA4a* expression increased in *phyB1B2*, compared to the WT (Figure 8A). In addition, *HSP90* gene was upregulated in *phyB1B2* compared to the WT and *phyA* (Figure 8B). These results clarified the relationship between the *phy* mutants and heat-responsive genes under HS conditions.

We also investigated the expression levels of other stress-responsive genes, such as *GLYCINE-RICH PROTEIN* (*GRP*) and *DEHYDRIN Ci7* (*DRCi7*). *GRP* plays a role in cellular stress responses and signaling. It has been indicated to be involved in the plant stress response in several plant species [60]. DEHYDRIN proteins are activated by various environmental factors [61]. *GRP* was highly expressed in *phyA*, with a significant difference compared to WT and *phyB1B2*. *DRCi7* expression level was higher in *phyB1B2* than in WT and *phyA* (Figure 8C). These results indicate that *GRP* may participate in *phyA* thermotolerant function.

PHYTOCHROME-INTERACTING FACTORS (PIFs) have roles in mediating heat responses in Arabidopsis [62]; therefore, we reviewed the expression levels of *PIF4* and *PIF7a*. The expression level of these genes in *phyA* and *phyB1B2* was similar to that in WT (Figure S5), which suggests that these genes did not participate in *phy* mutants’ thermotolerance.

Furthermore, we examined the expression levels of *SPEECHLESS* (*SPCH*), *9-CIS-EPOXYCAROTENOID DIOXYGENASE 1* (*NCED1*), *PROTEIN PHOSPHATASE 2C* (*PP2C*), *DELTA-1-PYRROLINE-5-CARBOXYLATE SYNTHASE* (*P5CS*), and *DELTA-1-PYRROLINE-5-CARBOXYLATE REDUCTASE* (*P5CR*) in WT and *phyA* and *phyB1B2* mutants at 37 °C.

SPCH is one of the basic helix–loop–helix (bHLH) transcription factors that lead to stomatal development [63]. Arabidopsis *SPCH* gene is necessary for asymmetric cell division regulation of stomatal lineage initiation [64]. The expression level of *SPCH* was downregulated in both *phy* mutants compared to WT plants (Figure S6A). This result indicates the difference in stomata development between WT and *phyA* and *phyB1B2* mutants.

The NCED enzyme controls the accumulation of ABA, which is an important plant stress-signaling hormone [65]. Furthermore, PP2Cs are involved in the ABA signaling pathway [66]. The expression level of *NCED1* and *PP2C* was enhanced in *phyB1B2* mutant compared to WT, but there was no significant difference between WT and *phyA* mutant (Figure S6B,C). According to these results, it appears that ABA plays a role in *phyB1B2* under HS.

P5CS and P5CR are the key enzymes for the proline biosynthesis pathway in plants [67]. The *phyA* mutant represented a higher expression of *P5CR* and *P5CS* genes compared to WT and *phyB1B2* mutant (Figure S6D,E). This result indicates that proline has a role in *phyA* mutant under HS.

#### 2.3.2. Relative Expression of Some Heat- and Stress-Responsive Genes as Well as Flower Controller Genes during the Flowering Stage

**Figure 9:**
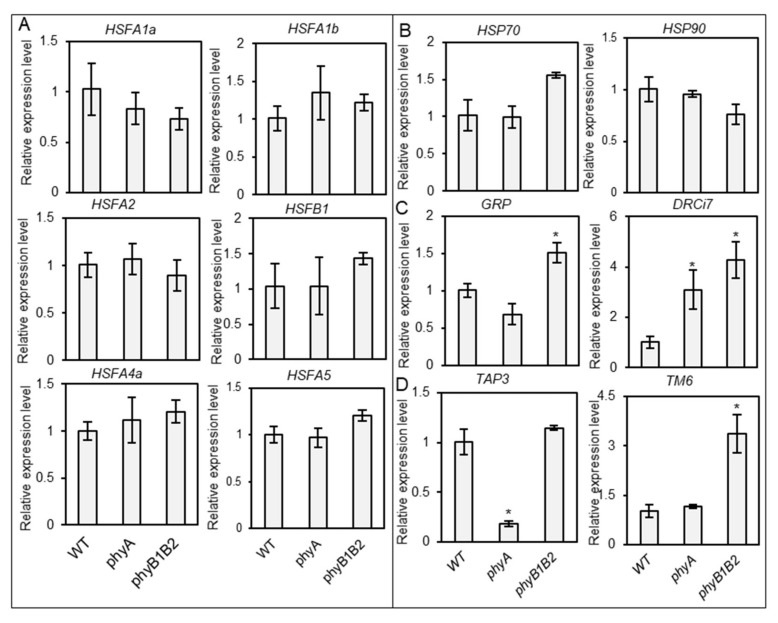
Relative gene expression level under heat stress (HS) during the flowering stage. The expression level of *HSFs* (*HSFA1a*, *HSFA1b*, *HSFA2*, *HSFB1, HSFA4a*, and *HSFA5*) (**A**), *HSPs* (*HSP70* and *HSP90*) (**B**), and stress-responsive genes (*GRP* and *DRCi7*) (**C**) of WT and *phyA* and *phyB1B2* mutants after one month under HS at 37 °C during the flowering stage. Flower-related genes (*TAP3* and *TM6*) (**D**) were observed after 35 days under greenhouse (GH) conditions when plants showed abnormal flower structures. The relative expression level of the WT was normalized to 1. Error bars represent standard deviation (*n* ≥ 3). The asterisk symbol (*) represents statistically significant differences between WT and *phy* mutants using Duncan’s test (*p* < 0.05).

After the initiation of flowering, we subjected plants to HS at 37 °C for one month to check the expression levels of *HSFs* and *HSPs,* as well as the stress-responsive genes *GRP* and *DRCi7*. The WT and *phy* mutants did not show any significant variation in the expression levels of *HSF* and *HSP* genes. *DRCi7* expression was significantly upregulated in *phy* mutants compared to that of WT. These results suggest that the heat- and stress-responsive genes did not play an obvious role during the flowering stage to promote heat tolerance in *phy* mutants (Figure 9A,B).

B-class MADS-box genes are involved in the specification of petal and stamen organ identity as well as in organ maturation control [68]. Tomato contains a single euAP3 lineage gene (*Tomato APETALA3* (*TAP3*)) and a single tomato MADS-box gene 6 lineage (*TM6*), which play distinct roles in floral development. *TAP3* is required to identify the petal and stamen, while *TM6* is important for stamen differentiation [69]. We investigated the relative expression levels of *TAP3* and *TM6* in sampled flowers after 35 days under GH conditions. *TAP3* was significantly downregulated in *phyA* compared to WT and *phyB1B2*, with no significant difference between them. *TM6* was highly expressed in *phyB1B2* compared to WT and *phyA*, without any marked difference between the last two varieties (Figure 9D). Based on these results, the floral development of *phyA* and *phyB1B2* mutant flowers was controlled differently than that of WT.

## 3. Discussion 

In our study, we screened the response of *phyA* and *phyB1B2* tolerance to high-temperature stress compared to the ‘Money Maker’ WT under different growth stages from seed germination to fruiting stage, and we observed the response of these mutants to HS during seed germination, vegetative growth, flowering, and fruiting stages.

Our findings demonstrated that tomato *phyA* and *phyB1B2* exhibited tolerance to HS during seed germination and the vegetative growth phase and for a short time of HS during the flowering stage. A long period of HS during the flowering or fruiting stage returned a response similar to that of the WT, which showed a sensitive response under all growth phases.

For the seed germination stage, both *phy* mutants showed nonsignificant differences in seed germination rate under normal and HS conditions, whereas the WT germination rate was negatively affected by HS, which indicates that both *phy* mutants can grow in hot seasons (Figure 1A).

During the vegetative growth stage, *phyA* and *phyB1B2* exhibited high tolerance to HS by enhancing the plant mechanism to cope with high-temperature stress. Inducing damage to plasma membranes in plants is an important side effect of HS [70]. Tomato *phyA* and *phyB1B2* exhibited lower EL compared to WT under HS (Figure 5A), even though the tomato ‘Money Maker’ is classified as having moderate heat tolerance [71], indicating that tomato *phyA* and *phyB1B2* are highly tolerant mutants of HS that enhance membrane thermostability. Increased leaf temperature is a result of HS, which in turn inhibits the activity of enzymatic antioxidants and considerably increases the MDA level in the leaves [72,73]. Tomato *phyA* inhibited the production of MDA under HS (Figure 5B), which could be an indicator of the inhibition of polyunsaturated fatty acid peroxidation in the cells and the reduction in oxidative damage to membrane lipids, which then enhance heat tolerance. The accumulation of osmoprotectants is an adaptive mechanism in plants against environmental stresses such as heat tolerance [74] and increases plant survival by protecting the cellular structure [75]. Under high temperatures, plants accumulate different osmolytes, such as proline [76]. Tomato *phyA* enhanced proline accumulation under HS conditions via enhancing the expression of *P5CS* and *P5CR* genes that control proline biosynthesis, suggesting that proline accumulation is involved in the heat tolerance of *phyA* (Figure 5C and Figure S6D,E).

In addition, under the vegetative growth stage, both *phy* mutants exhibited a reduced number of stomata compared to the WT under HS (Figure 6A,D), and *phyA* exhibited a decrease in stomatal aperture (Figure 6C,F). In Arabidopsis, *spch* mutant did not produce stomata or lineages [64]. Tomato *phyA* and *phyB1B2* mutants showed a downregulation in the expression level of *SPCH* gene compared to WT plants (Figure S6A), which may be one of the reasons for the stomata number reduction in *phy* mutants. It is plausible that water preservation also enhanced the heat tolerance of *phyA* and *phyB1B2*. Decreased stomatal closure and accelerated water loss speed enhanced HS sensitivity in transgenic OsMDHAR4-overexpressing rice [77]. Moreover, ABA is a key molecule that activates plant reactions to stress conditions, such as HS. It induces the accumulation of different proteins involved in stress acclimation and regulates stomatal closure under HS conditions [78].

Furthermore, the basic network among HSFA1a, HSFA2, and HSFB1 activity controls the HS response in tomatoes [79]. In addition, HSP accumulation protects the cell system from HS and enhances several functions and mechanisms to cope with HS [80]. In tomato *phyB1B2,* the upregulation of *HSFA2, HSFB1, HSFA4a,* and *HSP90* expression levels may enhance thermotolerance (Figure 8A,B). The results showed that the expression levels of *HSFA2* and *HSFB1* were upregulated in *phyA* (Figure 8A). HSFA2 is an ideal transcriptional activator during the HS response [81]. Meanwhile, *HSFA4a* is involved in the regulation of abiotic stress tolerance, such as salt tolerance in *Arabidopsis* [82] and cadmium tolerance in wheat and rice [83]. GRP is involved in the defense system of plants under biotic and abiotic stresses and has a high glycine content [84]. Tomato *phyA* showed a high expression level of *GRP* under HS (Figure 8C), which might play a role in heat tolerance.

HS negatively affects pollen development and fertility, which reduces fruit setting [85]. Over a short period (less than 14 days) under fluctuating high temperature in the GH, *phyA* exhibited a significantly enhanced percentage of developed flowers compared to WT and *phyB1B2* (Figure 4B). However, *phyB1B2* produced fertile pollen for a longer time under HS, followed by *phyA*, in comparison with WT pollen, which quickly reacted to high temperatures (Figure 7). Stigma exertion and antheridia cone splitting are floral characteristics used to evaluate HS tolerance [45]. *PhyA* achieved a significantly lower percentage of stigma-exerted and abnormal flowers compared to WT within 2 weeks under fluctuating high temperature; at the same time, no marked difference occurred when compared to *phyB1B2* (Figure 4C, D). Although *phyA* showed heat tolerance by floral characteristics during the early exposure period to HS, its abnormal floral structure was enhanced by long HS application during the flowering phase (Figure 2A). In tomato, the homozygous mutation in *TAP3* showed a classic B-class gene loss-of-function phenotype, which formed a complete transformation of the petals into sepaloid structures and the stamens into carpel-like organs [69]. This might explain the conversion in the flower structure of *phyA* due to the downregulation of *TAP3* by showing a lower expression level compared to WT and *phyB1B2* after exposure to HS for 35 days (Figure 9D). The long sepals were confirmed by the results of fruit calyx, which significantly increased in *phyA* compared to WT and *phyB1B2* (Figure 4F). In addition, in *phyA*, the enhanced heat-responsive genes and *GRP* that were upregulated during the vegetative growth stage did not show a marked difference compared to WT under long periods of HS during the flowering stage. The *phyB1B2* showed a nonsignificant variance in the expression level of all studied heat-responsive genes during the long exposure to HS during the flowering stage (Figure 9A,B). These results confirm that *phyA* and *phyB1B2* did not enhance HS tolerance during the flowering stage when exposed to a long period of high-temperature stress, as did the WT.

In tomatoes, small parthenocarpic fruit is one of the side effects of high temperature, which leads to a decrease in yield [86]. All observed fruit in WT plants and *phy* mutants were parthenocarpic, and the average FW of the fruits was low, indicating that *phyA* and *phyB1B2* did not enhance HS tolerance during the fruiting stage as observed with WT.

**Figure 10:**
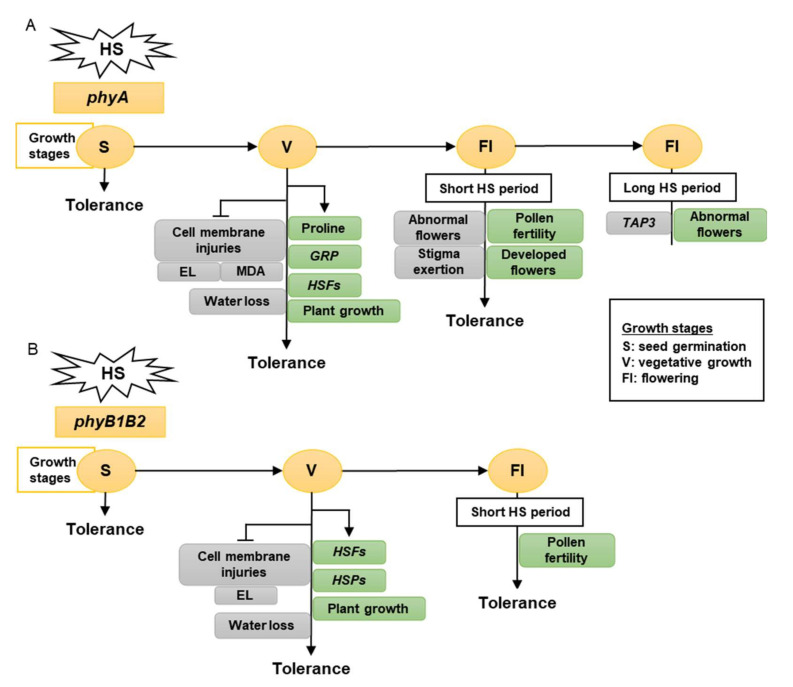
The model shows the response of *phyA* (**A**) and *phyB1B2* (**B**) mutations at different growth stages under heat stress (HS). The gray rectangles show the factors inhibited while the green rectangles illustrate the factors enhanced by stress application. The growth stages that were shortened in S, V, and Fl were the seed germination, vegetative growth, and flowering stages, respectively. The *phy* mutants exhibited tolerance to HS during S, V, and Fl stages. In S stage, the seed germination rates of both *phy* mutants were not significantly affected by HS compared to control conditions (A, B). In V stage, *phyA* enhanced the proline production, *HSFs*, *GRP*, and plant growth (A), while *phyB1B2* enhanced the upregulation of *HSFs* and *HSPs* as well as plant growth (B). In addition, both *phy* mutants inhibited cell membrane injuries (A, B). In Fl stage, *phy* mutants enhanced pollen fertility for a longer time compared to WT (A, B). Moreover, *phyA* exhibited an increase in the percentage of developed flowers and an inhibition in the percentage of abnormal and stigma-exerted flowers compared to WT. During a long HS application in the Fl stage, *phyA* exhibited enhanced abnormal flower formation via downregulation of *TAP3* which enhanced sepal and petal conversion (A).

Taken together, tomato *phy* mutants have a different response to high-temperature stress, as determined by the growth stage. The vegetative growth stage had the greatest growth, showing a high HS tolerance. Therefore, tomato *phyA* is a thermotolerant mutant that enhances membrane stability, proline accumulation, and water preservation by reducing stomata number and stomatal aperture and upregulating the expression levels of *HSFA2, HSFB1,* and *GRP*. In addition, it inhibited MDA accumulation in the leaves (Figure 10A). Tomato *phyB1B2* is a heat-tolerant mutant that enhances membrane stability and expression levels of *HSFA2, HSFB1, HSFA4a,* and *HSP90*. In addition, it inhibited water loss by decreasing the number of stomata (Figure 10B).

Finally, using plant genome editing on *PHY*A or B genes in other plant crops could be a good method of enhancing crop tolerance to HS. The genome editing method is a powerful technique for producing varieties; thus, several genome-edited plants are currently being produced [87,88]. Mutations in *HSFA6a* and *HSFA6b,* created by CRISPR/Cas9, are more tolerant to abiotic stresses such as ABA, mannitol, and sodium chloride [89]. Researchers have developed several mutants, such as the tomato *ead1* mutant and soybean *hsp90A2* mutant, using CRISPR/Cas9. The tomato *ead1* mutant exhibited short roots and increased sensitivity to ABA [90]. The soybean *hsp90A2* mutant is sensitive to HS, has decreased chlorophyll content, and has high levels of lipid peroxidation [91]. Generally, genome editing techniques introduce mutations in the targeted genes. Mutations in negative regulators enhance tolerance to abiotic stress. Thus, *PHYA* and *PHYB1B2* are good target genes for enhancing abiotic stress tolerance.

## 4. Materials and Methods

### 4.1. Plant Materials and Growth Conditions

We used the tomato (*Solanum lycopersicum* L. ‘Money Maker’) WT, *phyA* mutant, and *phyB1B2* double mutant [92] to study the role of tomato *PHYs* A, B1, and B2 in HS at the stages of seed germination, vegetative growth, flowering, and fruiting. We grew the seeds in soil or rockwool cubes and incubated them at 25 °C for a long-day photoperiod (16 h light/8 h dark).

Plants endured HS under two different conditions: stable high temperature at 37 °C or fluctuating high temperature under GH conditions. In the GH, the minimum temperature recorded was approximately 20 ± 3 °C during the night, while the maximum was approximately 57 ± 3 °C in the daytime during the summer seasons of 2019, 2020, and 2021 in Tsukuba City, Japan (Figure S1). We recorded GH temperature and humidity during the stress period using a Fujita Watch-Logger (KT-255F, Fujita Electric Works, Ltd., Kanagawa, Japan).

The plant ages differed when we applied HS according to the target growth stage to check the response of the *phy* mutants to HS conditions. To check the vegetative growth stage, we exposed one-month-old plants to HS, while for the flowering and fruiting stages, we transferred the plants to HS conditions after flowering.

### 4.2. Phy Responses to HS during the Seed Germination Stage

#### Seed Germination Rate

We incubated the seeds in wet tissue at 25 and 37 °C for one week and calculated the germination percentage using the following formula: seed germination rate = (number of germinated seeds/total number of seeds) × 100.

### 4.3. Phy Responses to HS during the Vegetative Growth Stage

#### 4.3.1. Morphological Phenotype

We measured the morphological phenotypic characteristics, such as root surface area, root length, stem height (from the soil surface), stem thickness, plant FW, leaf number/plant, and leaf FW of tomato plants.

To analyze the root surface area, we viewed the image data using the ImageJ software version 1.51 (https://imagej.nih.gov/ij/download.html).

#### 4.3.2. EL

We analyzed the EL of tomato leaves as previously described [93]. Briefly, we washed the leaf surface with Milli-Q water (MQ) to remove any ions from the surface and then flooded the leaves in a tube filled with MQ to cover all the leaf parts. The tube stayed in a hot water bath at 43 ± 1 °C for 1 h. When the temperature decreased to 20–25 °C, we measured the ionic conductivity 1 (C1), followed by autoclaving the samples at 121 °C for 10 min and re-examining the ionic conductivity 2 (C2) when the samples cooled to 20–25 °C. We used a conductivity meter (Lutron Electronics Co., Inc., Upper Saucon Township, PA, USA) for the ionic conductivity measurements and calculated the EL percentage using the following formula: EL (%) = C1/C2 × 100.

#### 4.3.3. Measuring Proline and MDA Levels

Proline content of the leaf samples was determined as described [94], and a DU-800 spectrophotometer was used to measure the absorbance at 520 nm (A_520_) (Beckman Coulter, Inc., Brea, CA, USA) for the calculation of the proline level using the following equation:

(1) $$\begin{array}{cc} \mathrm{Proline}\;\left(µ\mathrm{mol}/g\right) & \\ = & \frac{A\;520\;\left(µg\;\mathrm{proline}\mathrm{mL}\right)\times \mathrm{Toluene}\;\mathrm{amount}\left(\mathrm{mL}\right)}{115.13}\\ / & \frac{\mathrm{Sample}\;\mathrm{FW}\left(g\right)}{5} \end{array}$$

We measured the MDA level as described [95] and calculated it using the following formula: MDA (μmol/L) = (6.45 × (A_532_-A_600_) – 0.56 × A_450_).

#### 4.3.4. Microscopic Analysis for Stomata

To prepare the leaf samples for checking stomata conditions, we collected fresh leaflets and pasted a thick tape on the upper surface, which we then gently pulled from the leaflet to tear off the epidermis to access the thin transparent layer of surface cells. After placing the epidermis layer on a microscope slide, we cut the leaf using a sharp scalpel, added one drop of water, and placed a coverslip on the sample. We used an Olympus BX50 microscope (Olympus, Tokyo, Japan) to determine the stomata number, pore length, and aperture, with the first item under 40× magnification (counting area 92.7 mm^2^) and the latter two under 100× magnification.

#### 4.3.5. RNA Isolation and Quantitative RT-PCR

To check the heat- and stress-responsive genes during the vegetative growth stage, we extracted total RNA from the leaves of one-month-old plants of WT and *phy* mutants after exposure to HS at 37 °C for two weeks, while for the flowering stage, we used leaf samples from flower-initiated plants after exposure to HS at 37 °C for one month. For the expression levels of *TAP3* and *TM6*, we used flower samples from flower-initiated plants after exposure to HS under GH conditions for 35 days.

We extracted the total RNA from WT and *phy* mutant plant samples using TRIzol reagent (Thermo Fisher Scientific, Waltham, MA, USA) according to the manufacturer’s instructions and used 2 µg to synthesize cDNA using a high-capacity cDNA reverse transcription kit (Thermo Fisher Scientific, Waltham, MA, USA). The primers used for the real-time PCR are listed in Table S1. RT-PCR amplification and detection were carried out using THUNDERBIRD SYBR qPCR Mix (Toyobo, Osaka, Japan) running on a 7900HT real-time PCR system (Applied Biosystems/Thermo Fisher Scientific, Waltham, MA, USA). The relative transcript abundance calculation utilized the comparative C_T_ method as described previously [96] using ΔC_T_ of WT under HS as a subtrahend factor in the ΔΔC_T_ subtraction formula for comparison with the *phy* mutants as follows: ΔΔC_T_ (ΔC_T_ − ΔC_T, WT (stress)_). The tomato *EXPRESSED* gene was an endogenous control for gene expression analyses [97].

### 4.4. Phy Responses to HS during Flowering and Fruiting Growth Stage

#### 4.4.1. Pollen Fertility and Pollen Tube Growth Test

To extract the pollen grains, we collected newly bloomed flowers (7–10 flowers) from plants every day under GH conditions, isolated the anther cones from the flowers, and left them to dry for 3–4 h. We divided the anther cone into 2–3 parts and remove the pollen grains by knocking on the cones. Eppendorf 1.5 mL tubes held the pollen for microscopic analysis of pollen fertility and pollen tube growth.

To analyze pollen fertility, we stained the pollen with iodine potassium iodide (IKI) as described previously [98] and observed it using an Olympus BX50 microscope. We manually counted at least four different microscopic sections of the fertile pollen for each period using the Microsoft Paint application (Microsoft windows, version 20H2 © Microsoft Corporation)

The day after emasculating the mother flowers one day before opening, they were manually cross-pollinated using the extracted pollen after stress treatment. After 24 h, we collected the pistils and immersed them in a fixing solution (consisting of 3:1 ETOH 100%:acetic acid 100%) for 12 h, followed by immersion in ETOH 75% for 6–8 h and transfer to a softening solution consisting of NaOH 5M for 12–16 h. We prepared the aniline blue working solution one day before use by diluting the aniline blue stock 0.01% (V:V) with K_2_HPO_4_ (0.1 M, pH 10) 10 times and conserving it at 4 °C in the dark overnight. We then transferred the pistils into aniline blue working solution for 24 h, placed them on a glass slide using glycerol 100% as a mounting agent, and strongly pressed the cover glass to flatten the sections. Observation of the sections utilized an Olympus BX50 UV microscope.

#### 4.4.2. Flower Characteristics

We determined the number of flowers/cluster, percentage of developed flowers/cluster, percentage of abnormal flowers/cluster, and percentage of stigma exertion/cluster for the flowers of WT and *phy* mutants under GH conditions.

#### 4.4.3. Fruiting Characteristics

The average fruit FW, average calyx FW, and parthenocarpic phenomena were observed to determine the response of WT and *phy* mutants to HS under GH conditions.

### 4.5. Statistical Analyses

We used analysis of variance test (ANOVA) to analyze the quantitative data, with the means compared by Duncan’s multiple range test (*p* < 0.05) or post hoc Tukey HSD test.

## Data Availability

Data is contained within the article or supplementary materials.

## Supplementary Materials

Supplementary materials can be found at https://www.mdpi.com/article/10.3390/ijms23031681/s1.
