# Supplementary material for: Functional Characterization of Tomato Phytochrome A and B1B2 Mutants in Response to Heat Stress
Source: Int J Mol Sci. 2022 Jan 31;23(3):1681. doi: 10.3390/ijms23031681 (PMC8835780; doi:10.3390/ijms23031681)
Supplement: Supplementary file 1 [file ijms-23-01681-s001.zip › Supplementary material (tables).pdf]

Table S1. The primer sequences used in the quantitative RT-PCR test to analyze the relative expression levels of stress-and heat-responsive genes, as well as flower structure-controlling genes.

Target gene	Sequence (5'-3')		Reference
SlHSFA1a	Forward	GTCGAGGTTGGGAAATTTGG	
	Reverse	TGTTGTCTGAGCCTAACCAG	
SlHSFA1b	Forward	CAACAACTTCTCCAGCTTCG	
	Reverse	TAAGAAATCCCTCGTTCGCA	
SlHSFA2	Forward	CCAGTGCTACAGGAAGTGAT	
	Reverse	TTCATTGTCCATTGCAGCAG	
SlHSFB1	Forward	GTGGTGGACGTGGTAAAATG	
	Reverse	TACTCTGCTCCAACCTCAAC	
SlHSFA4a	Forward	TCCTTAACCAACGTGTCCAA	
	Reverse	TGGCAACCTTCTCTTCCTTT	
SlHSFA5	Forward	CACAGTCATAGTCACCCTCC	
	Reverse	TGGAGTTTTGCAGCAGATTG	
SlHSP70	Forward	TGCCCAAAGATGAGGTTGAT	
	Reverse	ATTCTGCTTGATTCTTCGCG	
SlHSP90	Forward	AGCGGTATTGGCATGACTAA	
	Reverse	TACGCAGAGTAGAAACCCAC	
SlGRP	Forward	CCGGAAGTGTTGTTCCTAGA	
	Reverse	CAATAAGCTCCAAAAAGCAGC	
SlDRCi7	Forward	TTGTGTTTCTGTGTTGTTTTGG	[1]
	Reverse	GCACATACATATGCACTTACATACAG	
SlTAP3	Forward	tcagacagaggatgggagaa	
	Reverse	agcttcagagaattgtccaca	
SlTM6	Forward	CTCCATTCGCAAATGCCACAGC	[2]
	Reverse	GAAGGCGAGGAACTTAGAAGAGCAA	
SlExpressed	Forward	GCTAAGAACGCTGGACCTAATG	[3]
	Reverse	TGGGTGTGCCTTTCTGAATG	
SlPIF4	Forward	TGGACCGAGACACATTTGAAC	
	Reverse	GGTCGCTGTAGATTGATTGC	
SlPIF7a	Forward	GCCACCACTAGTACTACCAC	
	Reverse	TCCATGTCCATCGATTGAGG	
SlSPCH	Forward	GGTGGAGGTGGTAATAGTGG	

	Reverse	GACGTTGTGGTTGTGATTGT	
SlNCED1	Forward	CAACAGCAAGGAAGAAGACG	
	Reverse	TGCCTCCAACTTCAAACTCA	
SlPP2C	Forward	TGCTAATGTCGGAGATTCTCGTGC	[4]
	Reverse	CTACTGCTAACTGCCCATCAACCC	
SlP5CS	Forward	CTTGCAAACTCAGTTCGTGT	
	Reverse	ATCCATCAGCAATCTCCGTT	
SlP5CR	Forward	GGTGTGGTTAAGTCTGGGAT	
	Reverse	GACTCAAAAGCAGTACGACG	

References

1. Miura, K.; Shiba, H.; Ohta, M.; Kang, S.W.; Sato, A.; Yuasa, T.; Iwaya-Inoue, M.; Kamada, H.; Ezura, H. *SlICE1* Encoding a MYC-Type Transcription Factor Controls Cold Tolerance in Tomato, *Solanum Lycopersicum*. *Plant Biotechnol.* **2012**, *29*, 253–260. https://doi.org/10.5511/plantbiotechnology.12.0303a.
2. Cao, X.; Liu, X.; Wang, X.; Yang, M.; van Giang, T.; Wang, J.; Liu, X.; Sun, S.; Wei, K.; Wang, X.; et al. B-Class MADS-Box TM6 Is a Candidate Gene for Tomato Male Sterile-1526. Theor. *Appl. Genet.* **2019**, *132*, 2125–2135. https://doi.org/10.1007/s00122-019-03342-z.
3. Choi, S.; Hoshikawa, K.; Fujita, S.; Thi, D.P.; Mizoguchi, T.; Ezura, H.; Ito, E. Evaluation of Internal Control Genes for Quantitative Realtime PCR Analyses for Studying Fruit Development of Dwarf Tomato Cultivar 'Micro-Tom.' *Plant Biotechnol.* **2018**, *35*, 225–235.
4. Sun, L.; Wang, Y.-P.; Chen, P.; Ren, J.; Ji, K.; Li, Q.; Li, P.; Dai, S.-J.; Leng, P. Transcriptional Regulation of SlPYL, SlPP2C, and SlSnRK2 Gene Families Encoding ABA Signal Core Components during Tomato Fruit Development and Drought Stress. *J. Exp. Bot.* **2011**, *62*, 5659–5669. https://doi.org/10.1093/jxb/err252.
