# Supplementary material for: Functional Characterization of Tomato Phytochrome A and B1B2 Mutants in Response to Heat Stress
Source: Int J Mol Sci. 2022 Jan 31;23(3):1681. doi: 10.3390/ijms23031681 (PMC8835780; doi:10.3390/ijms23031681)
Supplement: Supplementary file 1 [file ijms-23-01681-s001.zip › Supplementary materials.pptx]

## Slide 1
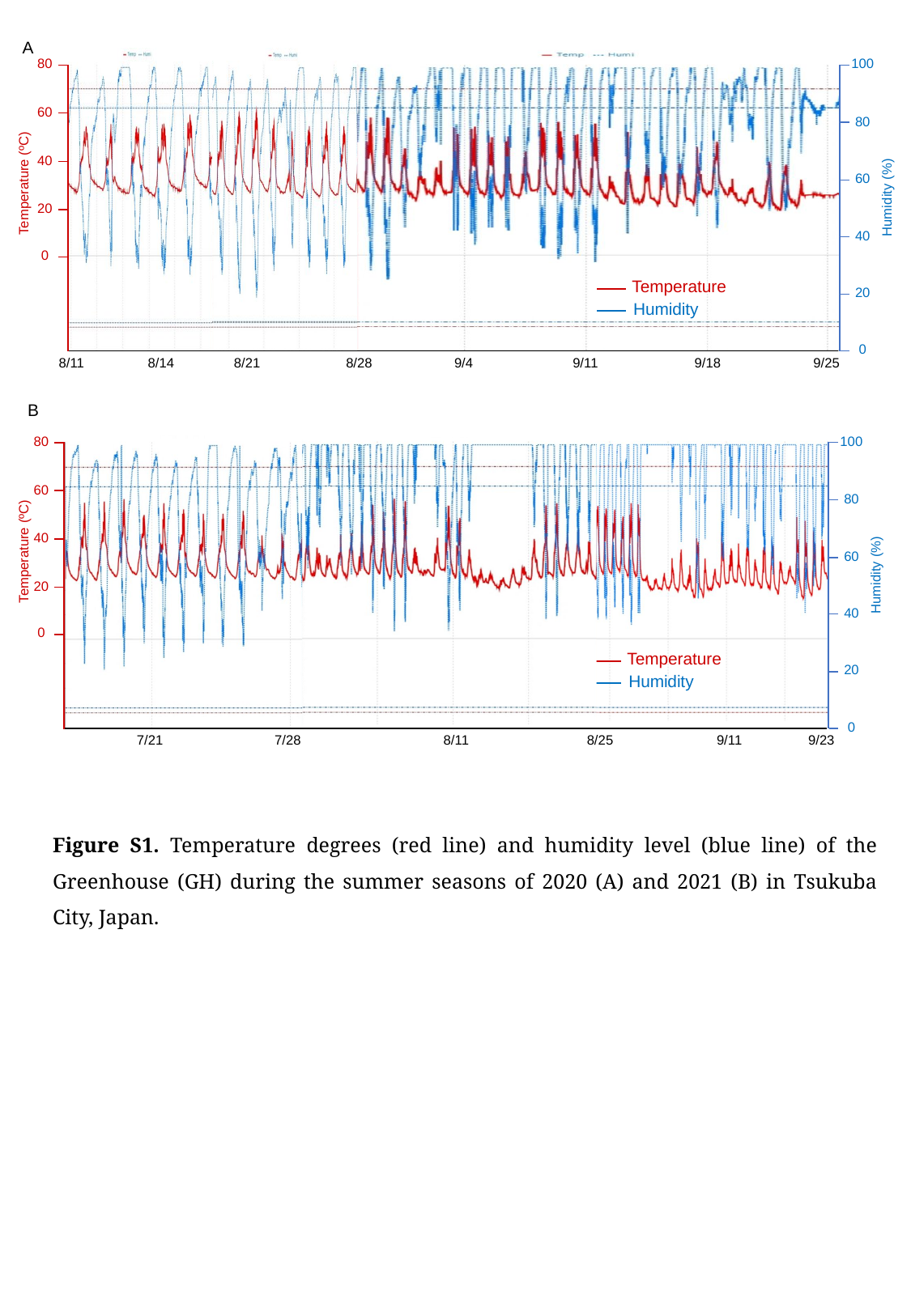

A
100
80
60
80
40
60
Humidity (%)
20
40
0
Temperature
20
Humidity
0
8/11
8/14
8/21
8/28
9/4
9/11
9/18
9/25
Temperature (ºC)
B
100
80
60
80
40
60
Humidity (%)
20
40
0
Temperature
Humidity
20
0
8/25
7/21
7/28
8/11
9/11
9/23
Temperature (ºC)
Figure S1. Temperature degrees (red line) and humidity level (blue line) of the Greenhouse (GH) during the summer seasons of 2020 (A) and 2021 (B) in Tsukuba City, Japan.

## Slide 2
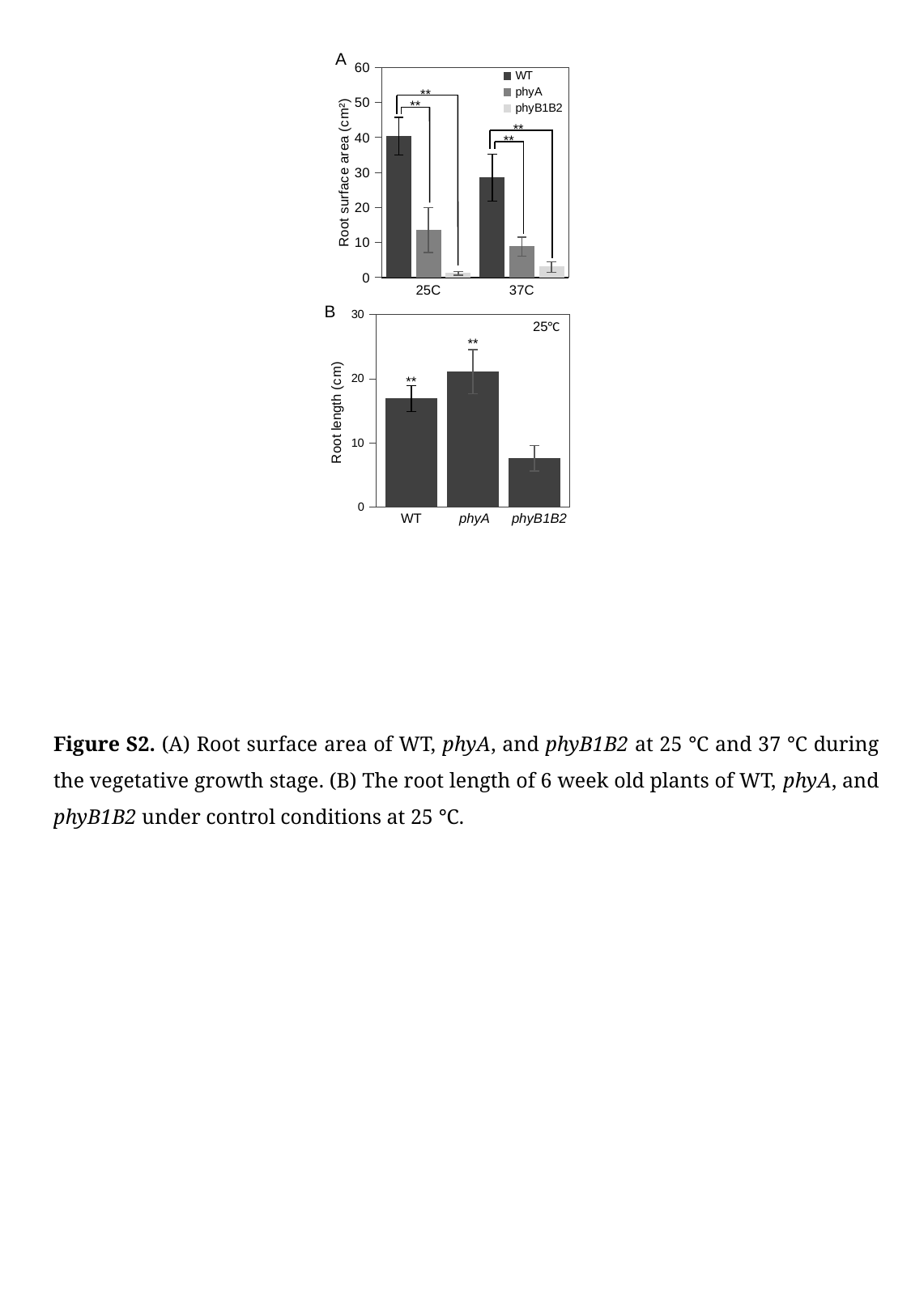

A
### Chart
| Category | WT | phyA | phyB1B2 |
|---|---|---|---|
| 25C | 40.405 | 13.537333333333335 | 1.165 |
| 37C | 28.514 | 8.765333333333333 | 2.97 |**
**
**
**
B
### Chart
| Category | WT | PhyA | PhyB1B2 |
|---|---|---|---|
| 25˚C | 16.9375 | 21.125 | 7.6 |**
**
WT
phyB1B2
phyA
25°C
Figure S2. (A) Root surface area of WT, phyA, and phyB1B2 at 25 ℃ and 37 ℃ during the vegetative growth stage. (B) The root length of 6 week old plants of WT, phyA, and phyB1B2 under control conditions at 25 ℃.

## Slide 3
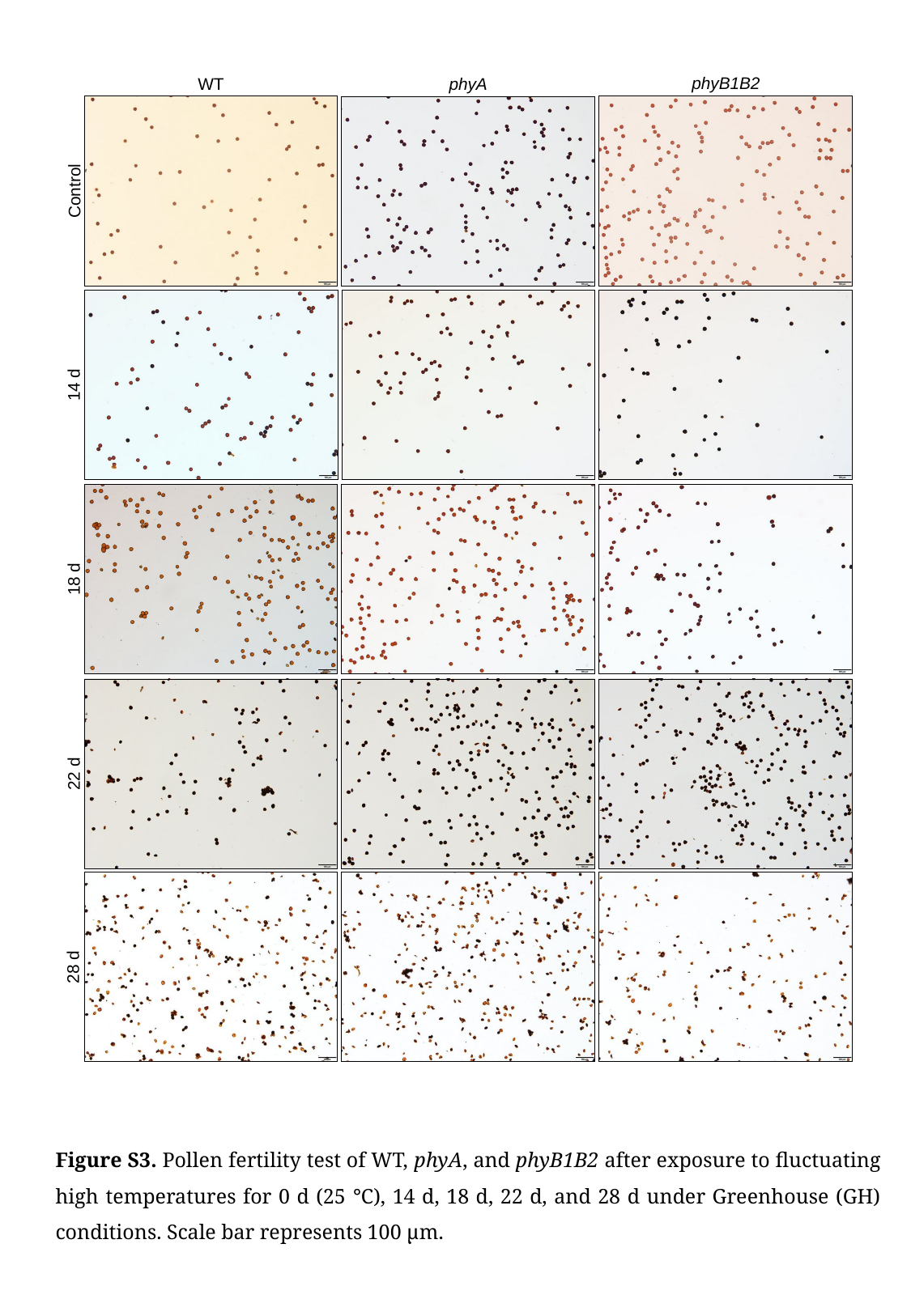

phyB1B2
phyA
WT
Control
14 d
18 d
22 d
28 d
Figure S3. Pollen fertility test of WT, phyA, and phyB1B2 after exposure to fluctuating high temperatures for 0 d (25 ℃), 14 d, 18 d, 22 d, and 28 d under Greenhouse (GH) conditions. Scale bar represents 100 μm.

## Slide 4
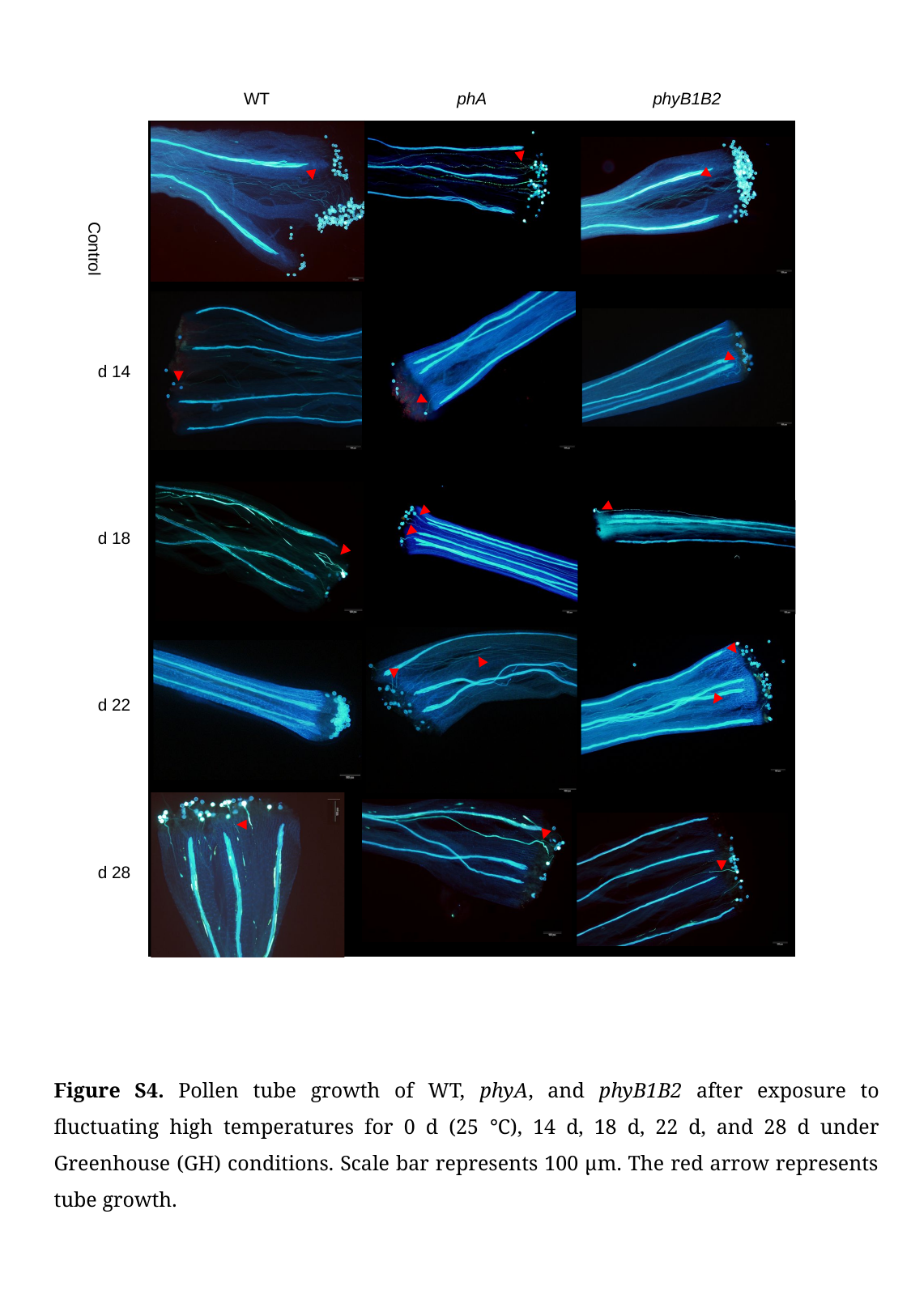

| | WT | phA | phyB1B2 |
| --- | --- | --- | --- |
| Control | | | |
| 14 d | | | |
| 18 d | | | |
| 22 d | | | |
| 28 d | | | |
Figure S4. Pollen tube growth of WT, phyA, and phyB1B2 after exposure to fluctuating high temperatures for 0 d (25 ℃), 14 d, 18 d, 22 d, and 28 d under Greenhouse (GH) conditions. Scale bar represents 100 μm. The red arrow represents tube growth.

## Slide 5
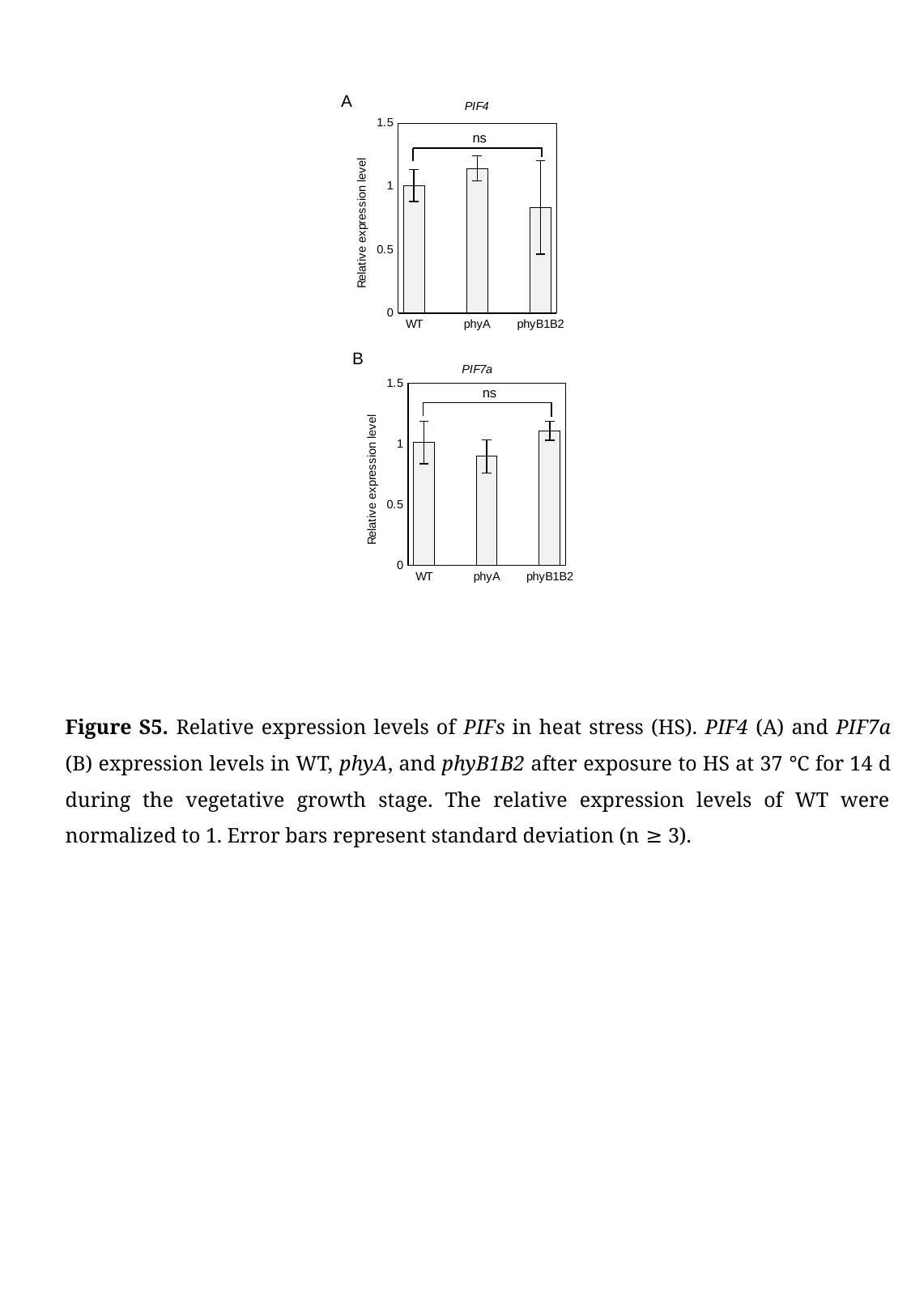

A
### Chart: PIF4
| Category | |
|---|---|
| WT | 1.006121642544818 |
| | None |
| phyA | 1.1413154211633274 |
| | None |
| phyB1B2 | 0.8320169059508669 |ns
B
### Chart: PIF7a
| Category | |
|---|---|
| WT | 1.0138063140251345 |
| | None |
| phyA | 0.899995955041008 |
| | None |
| phyB1B2 | 1.1111859074903738 |ns
Figure S5. Relative expression levels of PIFs in heat stress (HS). PIF4 (A) and PIF7a (B) expression levels in WT, phyA, and phyB1B2 after exposure to HS at 37 ℃ for 14 d during the vegetative growth stage. The relative expression levels of WT were normalized to 1. Error bars represent standard deviation (n ≥ 3).

## Slide 6
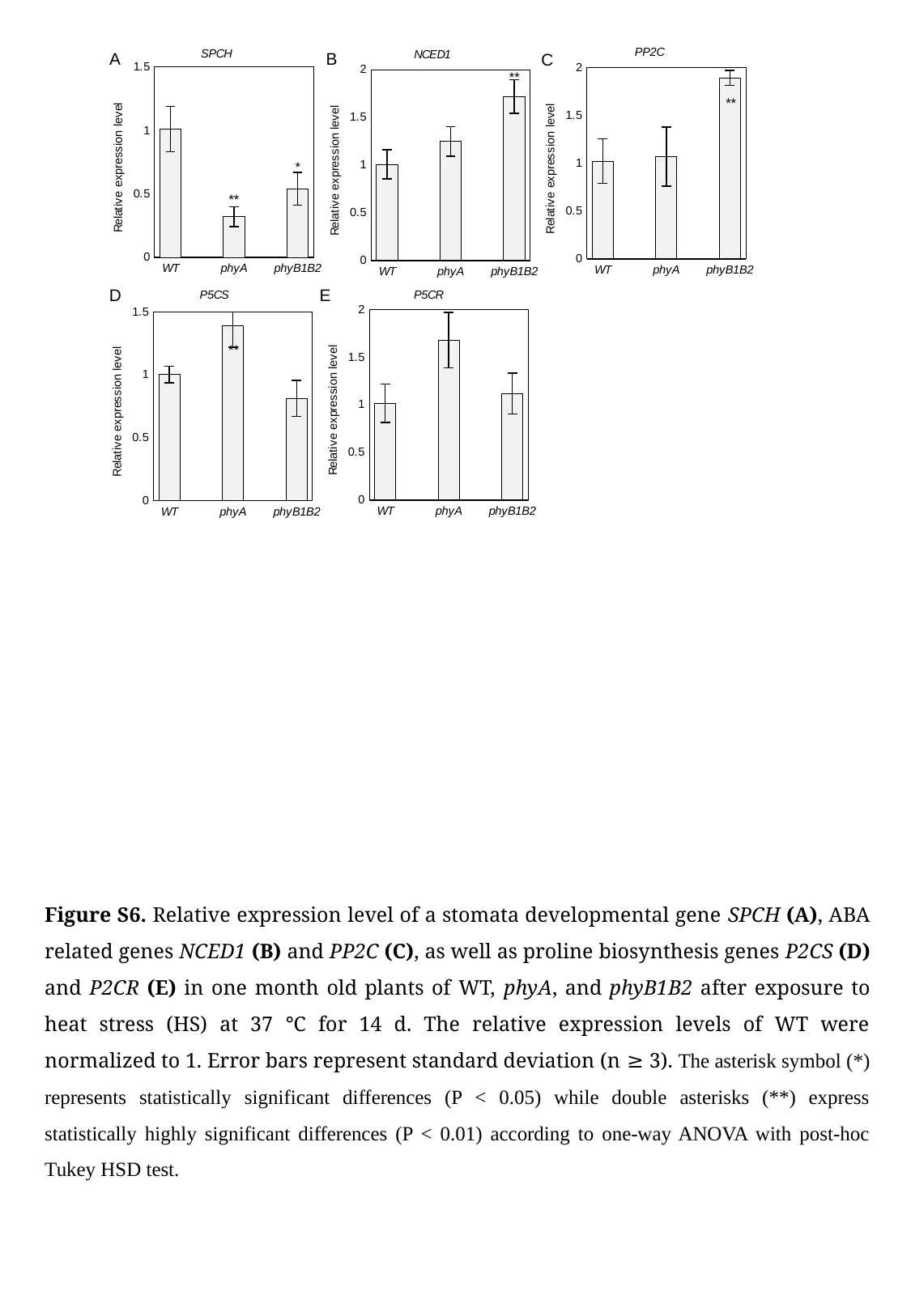

### Chart: SPCH
| Category | |
|---|---|
| WT | 1.011952897575302 |
| | None |
| phyA | 0.31984937629803034 |
| | None |
| phyB1B2 | 0.5401773610296317 |
### Chart: PP2C
| Category | |
|---|---|
| WT | 1.021765846794111 |
| | None |
| phyA | 1.0685937416131837 |
| | None |
| phyB1B2 | 1.8929545700963348 |**
C
B
### Chart: NCED1
| Category | |
|---|---|
| WT | 1.0086892258891733 |
| | None |
| phyA | 1.248961398472288 |
| | None |
| phyB1B2 | 1.7198879508177054 |**
A
*
**
D
### Chart: P5CS
| Category | |
|---|---|
| WT | 1.0017799397452207 |
| | None |
| phyA | 1.3871477135573882 |
| | None |
| phyB1B2 | 0.8129751162852706 |**
E
### Chart: P5CR
| Category | |
|---|---|
| WT | 1.0166369713284777 |
| | None |
| phyA | 1.678947146321388 |
| | None |
| phyB1B2 | 1.1181272541626441 |**
Figure S6. Relative expression level of a stomata developmental gene SPCH (A), ABA related genes NCED1 (B) and PP2C (C), as well as proline biosynthesis genes P2CS (D) and P2CR (E) in one month old plants of WT, phyA, and phyB1B2 after exposure to heat stress (HS) at 37 ℃ for 14 d. The relative expression levels of WT were normalized to 1. Error bars represent standard deviation (n ≥ 3). The asterisk symbol (*) represents statistically significant differences (P < 0.05) while double asterisks (**) express statistically highly significant differences (P < 0.01) according to one-way ANOVA with post-hoc Tukey HSD test.
